# The Development of the Infant

**Published:** 1890-01

**Authors:** 


### THE DEVELOPMENT OF THE INFANT.

From *Der Zeitschrift für Homöopathic.*

TRANSLATED BY A. McNEIL, M. D., SAN FRANCISCO, CAL.

Watching the mental development of her baby, as well as its physical growth, is to every mother a source of the tenderest solicitude, and also of the purest maternal joy, even from the first days of its existence. It is, however, not generally known at what periods of its age that its powers of understanding are awakened before it manifests by expression, gestures, and sounds that sensory perceptions have made an impression on it. W. Preyer, in his treatise "Die Seele des Kindes" (The Child's Soul) gives us reliable information, and it certainly is of interest to wider circles to learn the final results of his most pains-taking observations.

In the beginning of its existence the nursling is deaf, and it is at least a month before we can perceive, by its starting at sudden loud talking, that hearing has begun. Three to four weeks more pass before it has so far developed that usual sounds, such as singing, makes an impression. But not till the age of three months does it rise to clear consciousness of these sensory impressions, for, about this time, it begins to turn its head in the direction from which sounds have emanated.

The sense of sight is sooner developed. From the second to the fourth day the infant blinks if its eyes are touched by a bright beam of light, and after a week it can move its eyes independently, *i. e.*, without moving the head also toward the object. At this time, almost without exception, there is observed squinting, *i. e.*, the two eyes are turned in different directions; this disappears almost always when it is almost three months old, and again returns at thirty-six weeks when an object is placed close to its eyes. Also there occurs, regularly, to the end of the twelfth week, turning up of the eyes, the pupils are directed upward, and the upper lid falls lower, and only the white remains visible. At the end of this time, *i. e.*, the twelfth week, the sensitiveness of the optic nerves is fully developed. This is easily perceived by the rapid movement of the eye-lashes which is always seen when an object is brought quickly before the eyes, or when it is frightened. This is always accompanied by suddenly raising the arms. At this age (twelve weeks) it closes its eyes, when being bathed, in order to guard them from the water, while, during the first days and weeks of its life, the tepid water of the bath makes no impression on the open eyes.

The real power of the eyes in discerning is developed tolerably early—in the seventh week. Then the infant begins to fix its eyes on objects at the distance of a metre, and when they move its eyes follow them. Three weeks afterward the child's expression is more cheerful on seeing its mother's smiling face, brilliant or moving objects.

In three weeks more—that is, in the thirteenth week—it discriminates between light and darkness, which is easily seen by its expression when the dark room is lighted up. Between the seventh and eighth month it perceives its bottle at a distance of two to three metres, and at full nine months it manifests its desire for the bottle when it is being prepared, by pursing its lips and opening wide its eyes. When a year and a half old it tries to catch the smoke of a pipe or cigar, and of a jet of water. When two years old it has the ability to discern simple colors, but mixed colors not till after the fourth year; at this age its perception for forms begins, and it makes the first attempt to draw a circle.

Taste is sense which is first developed, and which continues through the period of nursing with the least change. Even during its earliest days the taste of sweets produces an expression of satisfaction, and it is preferred to that of any other.

Likewise an early development of the sense of smell may be shown, although not without exception, in the case of some nurslings. Many babies will not nurse the breast of a wet nurse who has an unpleasant odor from her person, but will immediately turn to another one. Whether a nursling recognizes its mother by the sense of smell, as in many young animals, is doubtful.

The sense of touch shows an early development. In a few days after birth the lips make the motion of sucking when touched. The tongue when touched also makes the motion of swallowing, and the palate as if retching, while the face reveals an expression of loathing. If the eyelids are blown upon or touched they close; the open eyes, however, as above mentioned, during the first weeks of life are not sensitive. The sensitiveness of the skin to mechanical influences, such as the pricks of pins, is present during the earliest days, and most clearly on the soles of the feet, palms of the hands, and the point of the nose. The very young infant shows its sensitiveness to temperature very plainly on the skin, and still more in its mouth. The sense of touch is indistinct till about the twenty-fifth week ; not till after this period does it begin to discriminate the objects it touches ; for instance, its own hand from that of another.

Every nursling, even if only a few weeks old, according to the development of its sensory perceptions, which receives sufficient attention, expresses its different frames of mind, arising from its comfort or discomfort, so clearly that it might be called voluntary if it had the power of voluntary expression. Until the child can speak it can only reveal its feelings by gestures, looks, or inarticulate sounds. And there are mothers and nurses not a few, who, on account of the lack of power of observing, or from thoughtlessness, often leave an infant to fret and cry a long time before they discover what is necessary to its comfort. Certainly, only by very careful observation of the manifold expressions of the emotions of the nursling can it be understood. However, no mother should neglect the effort to comprehend her child as soon as possible.

Of course it demands the most careful observation to differentiate the expression of discomfort, which may arise from very different causes. Displeasure, which is frequently observed even in healthy babies, is expressed by shutting the eyes tight, turning the head, change of the countenance, drawing down of the angles of the mouth, and crying. From the twelfth to the twentieth week ill humor is always manifested by a peculiar movement of the naso-labial fold, and in the same way during sleep; from the third month to the third year by the almost quadrangular shape of the mouth. The nursling does not groan or moan. When it cries, whether it is from pain or hunger is clearly seen by its sound. The cry from hunger is not constant, but is interrupted by longer or shorter intervals, and is not so high or violent as the cry of pain. The hungry baby shuts its eyes tight, while the tongue is broad and retracted into the mouth. If even with these indications, one is still at a loss to decide as to the cause of the crying, then if the breast or bottle is offered it is stopped immediately and it expresses satisfaction by a cooing sound.

During the first weeks of life the feeling of satisfaction after nursing is accompanied by fatigue in consequence of the exertion of sucking, and it usually sleeps. After the fourth week it indicates its satisfaction by cooing while its eyes are half open; after twenty weeks by turning away its head from the breast or bottle. When tired the nursling stops nursing and crying and loses its interest in singing and music. During the first month the infant sleeps sixteen hours a day and usually two hours at a time. In the second month, three to five; in the third, four to five; in the fourth, five to six, and sometimes even nine; in the sixth, six to eight. After the first year it sleeps thirteen, of which nine to ten are in the nighttime. In the third year, eleven to twelve of sleep in the night are sufficient without any in the daytime.

The nursling manifests joy or pleasure in many different ways. In the first three months it reveals its happiness by opening its eyes wide, which are brighter thereby, and by kicking, when it nurses, is pleased with its bath, when its diaper is changed or when it sees the loving face of its mother bending over it. After it has reached the age of twenty weeks, carrying into the open air gives visible pleasure. It also takes delight about this time in grasping with its little hands, tearing paper, seeing small animals, a watch, the ringing of a bell, and the pulling at some one's beard gives it great enjoyment. After the twenty-fourth week it manifests its joy by laughing, and after forty-four weeks by clapping its hands, and finally, somewhat later, it exults at its attempts to walk, when it is wrapped or unwrapped, when turning over leaves, when looking at pictures, etc.

There remains to be mentioned the expression of astonishment which is manifested at thirty weeks in opening wide its eyes and mouth; of fear, for instance, of small animals or certain things in the thirty-sixth week. Fear is an inherited, not an acquired emotion.

Hand in hand with this development of the sensory activity we have described goes the development of the will and understanding. These expressions of the will may be divided into reflex or involuntary in design or voluntary movements, and in imitation and expressive ones.

To the first category belongs retching and yawning, which is observed after the first week, and sighing, which occurs after the twenty-eighth week. Urinating is of like character, which the nursling of thirty-six to forty weeks distinctly announces, showing to the mother or nurse that the time has come to accustom it to cleanliness. Sucking is an instinctive movement which is inherited. Movements belonging to the second class usually develop in a definite succession. Starting from the first sensory perception, taste, what the infant tastes he wants to see. If it sees anything it desires to taste it, and as desire is in the baby only a wish to taste and this is increased when the object suits its gustatory appetite, it finally grasps after everything it sees and puts it in its mouth to taste until it learns that not everything which it gets is enjoyably, that many substances either do not taste at all or taste disagreeably. Until the eighth month the nursling fails in its attempts to grasp objects—*i. e.*, it does not reach far enough; after that time its movements are more certain, so that it is able in the eleventh month to bring its bottle to its mouth, for instance, and to take a hair from the table and examine it. Yet all of these are to be considered experimental attempts. Not before a year and a half has it so far developed in its faculties that it makes accurate attempts to seize objects with *one* hand, and after four months more it finally has both hands in complete command.

About the fourteenth week it begins to hold its head aright for a moment at a time, and fourteen days afterward it is so far under control that it no longer falls to one side or the other. In progressive development the will of the child gradually controls the whole body. It begins to sit up from the seventeenth to the twenty-sixth week. It repeats the attempts, sitting longer at a time, so that at the forty-second week it is capable of sitting with its back straight for moments at a time. At the forty-fourth week it is able to sit up at will, and at the same time the legs become straight, so that the soles of its feet no longer turn inwards. If it has accomplished the first effort to stand in the fortieth week, it will be observed to climb up by chairs and stand, and in the forty-fourth week its ability to stand has become greater, and while doing so it stamps its foot on the floor. The first attempts to walk are made in the forty-first week; till then all children creep. From the first essay at walking to complete success, the period is different in different children, clearly because not only the strength of will but physical power must both be considered, for the latter must correspond with the weight to be supported. Only a fifth of all children walk entirely alone before the first birthday; two-fifths more before the fourteenth or fifteenth month; another fifth cannot dispense with leading strings on the hand until from the sixteenth to the eighteenth, and the last fifth require a still longer time. But this physical ability for a long time only extends to walking in a straight line. More complex movements, such as turning around, climbing, jumping, or throwing, cannot be accomplished before the second or third year.

Imitative movements are only observed proportionately late, not before the twenty-eighth week, as these require not only the perceptive faculties of the child, but also its memory must be exercised, but, as we will show further on, this is active comparatively early, but only in a limited degree. So it is easily seen that young nurslings observe the movements of grown persons, but they are unable to retain these movements in their memory and imitate them, even if the physical powers are developed to an equal extent with the will. The expressive movements occur earlier, which in their effect express very nearly joy, pleasure, or pain, which have been already described. Smiling is observed in the seventh to the tenth week, when it has nursed, when a beam of light or music has pleased it. Laughter is seen in many children in the eighth week, and in others not before the seventeenth. From that time onward every nursling expresses its joy by laughter, which, week by week, becomes more lively and energetic till the twenty-fourth week ; it may change to a jubilant happiness, in which, as is often expressed, tears of joy stand in its eyes.

New impressions early call out voluntary expressions in the infant, as early as from the third to the seventh week. It may be that during sleep dreams busy its senses, and make an impression on the taste, vision, or hearing, or it may be that while awake it for the first time sees, hears, or feels something. And during this period it may be perceived that new impressions have been made on it. About this time, say in the fourth week, tears will flow while weeping as an expression of physical pain.

Finally, as to kissing. While the baby under a year allows itself to be kissed, and may, even by opening its mouth and inclining its head toward its mother, or the one in whose arms it is carried, show its desire to be thus caressed, yet not until it is fifteen months old is it fully conscious of the meaning of the act, and purses its lips when kissed.

As to the development of the understanding, this depends in a great measure on inherited ability. The first to show its activity is memory, which appears long before the first thirty weeks, yet its development extends only to those things which are closely related to its needs. For example, it knows how milk tastes and smells, and therefore detects immediately any other drink that is offered to it, which is clearly seen by its manner of taking it. Its feeling about when nursing, and searching around with its mouth in order to find the nipple, reveal the activity of memory. And before the time mentioned, it recognizes father, mother, and nurse. That it sees the persons around it may be observed in the second month, but neither its features nor movements indicate that it discriminates between them. At four months it perceives on awaking, when alone, the absence of its mother or nurse with displeasure and cries, and many an infant at this age will do so when its mother leaves the room. When it is five months, if its cloak and hood are shown it, or put on, it will manifest by its countenance or gestures that it wants to be taken out, and if this is not done it will cry. In the twelfth month it recognizes the hot stove or the flame of a candle, for it will, if brought near them, always try to keep back or turn aside.

The slowest sense to develop is speech, but we may observe in children mentally sound, that they understand well what is said to them before they are able to imitate the sounds, words, and syllables which they hear. Evidently the acquisition of language is influenced by the development of the memory. If it has a very early and high degree of perfection, so will speech come at an early age.

As soon as the infant succeeds in understanding the sounds of its future mother tongue, and before it really begins to speak, and is able to form the sounds correctly, it jumbles them all together of its own accord in all possible variations, and appears to derive great pleasure in making this babel of sounds. The order in which babies produce the different words is different in different individuals.

